# Objective Dynamic Assessment of Facial Movement Asymmetry in Children Using a Marker-Based Video Method

**DOI:** 10.3390/jcm15051870

**Published:** 2026-02-28

**Authors:** Dawid Danecki, Agata Sage, Zuzanna Miodońska, Sebastian Zowada, Anna Lipowicz, Andrzej Myśliwiec, Krzysztof Dowgierd, Ewa Piętka, Michał Kręcichowst

**Affiliations:** 1 Department of Medical Informatics and Artificial Intelligence, Faculty of Biomedical Engineering, Silesian University of Technology, 41-800 Zabrze, Poland; dawid.danecki@polsl.pl (D.D.); agata.sage@polsl.pl (A.S.); zuzanna.miodonska@polsl.pl (Z.M.); ewa.pietka@polsl.pl (E.P.); 2 Laboratory of Physiotherapy and Physioprevention, Institute of Physiotherapy and Health Sciences, Academy of Physical Education, 40-065 Katowice, Poland; sebas.zowada@interia.pl (S.Z.); a.mysliwiec@awf.katowice.pl (A.M.); 3 Department of Anthropology, Faculty of Biology and Animal Science, Wroclaw University of Environmental and Life Sciences, 50-375 Wroclaw, Poland; anna.lipowicz@upwr.edu.pl; 4 Head and Neck Surgery Clinic for Children and Young Adults, Department of Clinical Pediatrics, Collegium Medicum, University of Warmia and Mazury, 10-561 Olsztyn, Poland; krzysztofdowgierd@gmail.com

**Keywords:** facial movement analysis, facial asymmetry, video analysis, objective assessment, physiotherapy, biomedical signal processing, pediatric population

## Abstract

**Background**: Facial movement symmetry is an important indicator of neuromuscular function, with asymmetries associated with neurological disorders, trauma, and surgery. Quantitative symmetry assessment supports diagnosis, therapy monitoring, and surgical planning. This study proposes a marker-based approach to improve tracking stability and investigates whether dynamic facial movement descriptors can distinguish symmetric from asymmetric exercise execution. **Methods**: Videos were recorded using a low-cost acquisition setup during two facial exercises: eyebrow raising and smiling (75 patient; mean age 14 ± 4 years). Seventeen ArUco markers were placed at predefined facial landmarks. The dataset comprised 134 recordings labeled as symmetric (S) or asymmetric (AS). The processing pipeline included marker and face detection, symmetry axis estimation, feature extraction, and statistical analysis. Features were based on distances between paired markers and the estimated facial symmetry axis, yielding two dynamic descriptors: VertDist (vertical displacement) and Ratio (relative position across facial halves), along with their first derivatives. **Results**: Group differences between S and AS movements were analyzed using Welch’s *t*-test with effect sizes quantified by Hedges’ g. Statistically significant differences were found primarily in the first derivatives of VertDist and Ratio. For eyebrow raising, VertDist showed large effects (Hedges’ |g|=1.41−1.42) and Ratio moderate effects (|g|=0.75−0.87). For smiling, VertDist demonstrated moderate effects (|g|=0.87−0.93), while Ratio exhibited large effects (|g|=1.14−1.21). **Conclusions**: The proposed marker-based method enables reliable, low-cost quantitative assessment of facial movement asymmetry. Dynamic descriptors derived from VertDist and Ratio effectively differentiate symmetric and asymmetric facial movements.

## 1. Introduction

The facial muscles, innervated by the facial nerve, are responsible for performing precise, coordinated expressive movements, the correct execution of which requires the integrity of both the central and peripheral nervous systems, as well as the efficiency of the muscles themselves and the neuromuscular connections. From the point of view of anatomy and biomechanics, the muscles on the left and right sides should work symmetrically, although absolute symmetry is not required here [1]. The system is designed for patients undergoing treatment and rehabilitation for facial dysfunction (following orthognathic surgery, skin-muscle flap transplants, and central and peripheral nervous system paralysis). In fact, the goal of therapy is always to achieve symmetry, and obtaining objective information about the presence of differences allows for monitoring progress or lack thereof [2,3].

Many studies indicate that facial symmetry is a determinant of a person’s attractiveness and biological condition [4]. It is also true that symmetry does not directly translate into the perception of a person, and these relationships can be mediated by facial movements (e.g., caused by emotion) as well as markers of health [4]. Furthermore, symmetry is not a constant feature in either physiology or pathology and may change over time, including during treatment and rehabilitation [5]. Facial dysfunction, especially when the prognosis for full recovery is poor, can significantly affect self-esteem and the quality of social relationships [6].

Facial movement symmetry assessment is used, among other things, for diagnosing and monitoring rehabilitation for patients with facial nerve dysfunctions, evaluating the effectiveness of therapy, and planning and controlling the outcomes of surgical treatments [7]. The analysis of symmetry changes over time enables an objective assessment of therapy progress and facilitates a comparison of results between patients [8]. The use of facial symmetry assessment is also essential in speech therapy and neuro-speech therapy [9].

In clinical practice, facial expression symmetry is primarily assessed by visual observation and descriptive scales completed by specialists. These methods, despite their widespread use, are highly subjective and exhibit limited repeatability, making it difficult to accurately compare results across studies and centers [2].

To address these limitations, efforts are underway to objectify facial expression assessment using computer-aided diagnostic (CAD) methods and automated image processing techniques. Literature describes solutions based on facial geometry analysis, landmark tracking, and the use of machine learning-based tools such as MediaPipe or other facial landmark detection libraries [10,11,12].

Despite the dynamic development of markerless and machine-learning approaches, their use in clinical setting is limited. Recent methods demonstrate high accuracy and repeatability in the specified conditions, their performance can vary in less standardized environments. Variability in lighting, different head positioning, and acquisition protocols with differences between recording sessions, still pose challenges for consistent landmark detection and longitudinal assessment. Importantly, these limitations are increasingly addressed at the methodological level but their translation into robust, standardized clinical workflows remains an open challenge [2,8,13].

In this study, we applied a marker-based approach using physical reference points to improve tracking stability and measurement repeatability in facial movement analysis. In our research, we assumed that the dynamic parameters of facial movements allow for a reliable differentiation of symmetrical and asymmetrical performance of facial muscle exercises. The goal of this study is to quantitatively analyze the functional asymmetry of facial movements using objective features derived from video recordings.

From a clinical perspective, the proposed method has the potential to support future research on patients with facial nerve dysfunctions, including those resulting from trauma or surgical interventions. By providing an objective and repeatable quantification of facial movement symmetry the approach may serve as a quantitative research tool for objective assessment of facial movement in controlled clinical studies [14,15]. However, further validation is required to establish diagnostic performance, population reference values, and relations with established clinical grading scales before clinical application. By standardizing measurements and ensuring their repeatability, this tool can facilitate comparison of outcomes across patients and clinical centers, contributing to more effective and personalized clinical care [16].

## 2. Materials and Methods

### 2.1. Video Material

This study used a custom-developed measurement setup and a dataset of facial video recordings acquired according to a unified data acquisition protocol.

#### 2.1.1. Measurement Station

We developed a low-cost measurement station enabling data acquisition under repeatable conditions. It consisted of a photographic backdrop, a ring light, an adjustable tripod, and a camera recording video at 4 K resolution (50 fps).

The distance between the camera and the patient was constant across all recordings (30 cm ± 5 cm), and participants were instructed to maintain a neutral head position.

Seventeen ArUco markers were placed at predefined anthropometric facial landmarks on each patient’s face, with one marker serving as a reference point. The described measurement setup had previously been used in a pilot study, the results of which were presented in an earlier publication [17].

#### 2.1.2. Data Collection

The measurement station was used to record video of patients performing two facial exercises, enabling assessment of functional symmetry.

Individuals with craniofacial dysfunctions were eligible for the study, including patients who had undergone orthognathic surgery, those with genetic defects, and those undergoing oncological, otolaryngological, or neurological treatment. A necessary condition for participation was full cooperation during the video recording and written consent from the child’s legal guardian. Each patient was instructed by a physiotherapy specialist and performed two exercises: “Eyebrow raising” and “Smiling” (performed independently for 20 s each).

The study included 75 children receiving treatment at the Department of Reconstructive and Aesthetic Maxillofacial Surgery of the Regional Specialist Children’s Hospital (Olsztyn, Poland). The mean age of the study group was 14±4 years. The reasons for treatment among the examined participants were craniofacial disorders diagnosed by a dentist specializing in maxillofacial surgery, including dysfunctions of the stomatognathic system, genetic defects, neoplastic disease, otolaryngological conditions, and neurological disorders. Patients with these dysfunctions were intentionally included; however, the study aimed not to diagnose pathologies but to demonstrate the potential applications of the proposed system in this clinical context.

Our database contained video recordings from 75 patients; Table 1 presents the number of patients qualified for further analysis, divided into symmetric (S) and asymmetric (AS) executions. The final dataset used in our experiments consisted of 134 recordings: 63 eyebrow-raising and 71 smiling videos. We excluded the remaining data due to low quality, primarily caused by partial marker occlusion that impaired accurate detection. More eyebrow-raising trials were excluded due to hair covering the facial markers; however, this did not require excluding participants, as their smiling recordings remained usable.

Our research received approval from the Bioethics Committee of the Jerzy Kukuczka Academy of Physical Education, Katowice, Poland (approval no.: 1/2021; 28 October 2021). Prior to the study, written informed consent was obtained from the legal guardians of all participants for participation in the study as well as for video recording and analysis.

### 2.2. Methods

**Figure 1:**
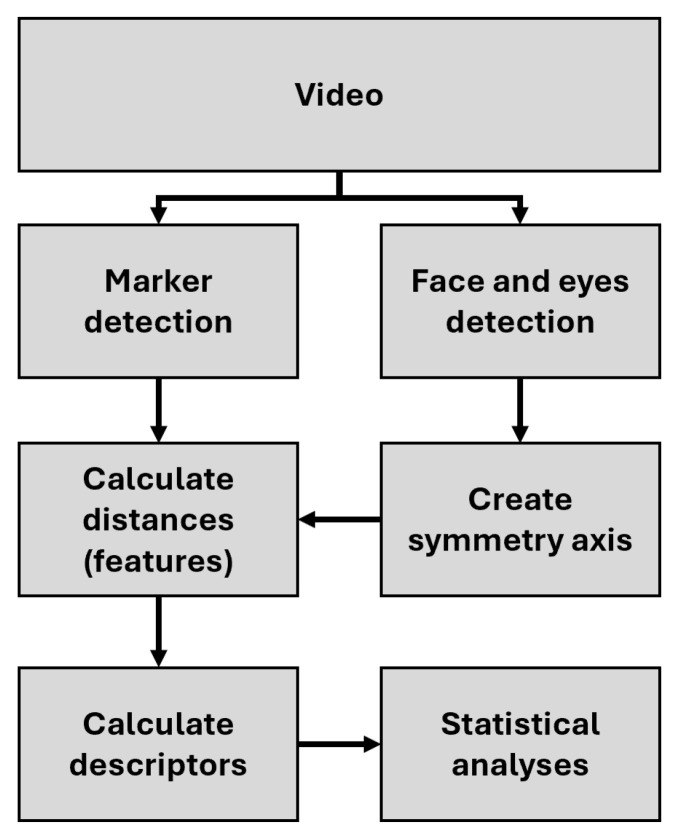
Overview of the analysis pipeline used for marker-based facial movement assessment.

The analysis pipeline (Figure 1) consists of video acquisition, marker and face detection, symmetry axis estimation, feature extraction, and statistical analysis.

The first stage of video processing involved automatic marker detection and tracking. A detailed description of the applied marker detection and tracking method, error filtering, and value conversion to metric units was presented in our previous work [17].

Then, we performed an automatic face detection in each video frame using a cascade classifier [18,19]. Face detection was used as a preliminary step to detect eyes and estimate the symmetry axis in each video frame. In the subsequent step, we detected the left and right eyes within the identified facial area.

Based on the detected eye regions, their centroids were determined. To ensure continuity across consecutive video frames, linear interpolation was applied when single-frame detection failed.

The obtained eye centroid coordinates were subjected to a filtering procedure to remove outliers resulting from single incorrect detections. Artifacts were defined as non-physiological changes in centroid position between consecutive frames. To make the criterion independent of image resolution and facial scale, the detection threshold for such jumps was expressed as a relative unit and set to 10% of the interocular distance (IPD) in each frame. Exceeding this threshold was classified as a detection error. In such cases, the coordinates were replaced by the average of the two neighboring frames, which limited abrupt displacements while preserving temporal continuity of the trajectory.

We then used the filtered eye-centroid trajectories to estimate a two-dimensional dynamic facial symmetry axis. For each video frame, we connected the centroids of the left and right eyes to form a line segment, computed its midpoint, and constructed a line perpendicular to this segment passing through the midpoint. This perpendicular line was assumed to represent the facial symmetry axis for the given frame. The proposed symmetry axis should be interpreted as a geometric, rather than functional, descriptor of facial symmetry.

**Figure 2:**
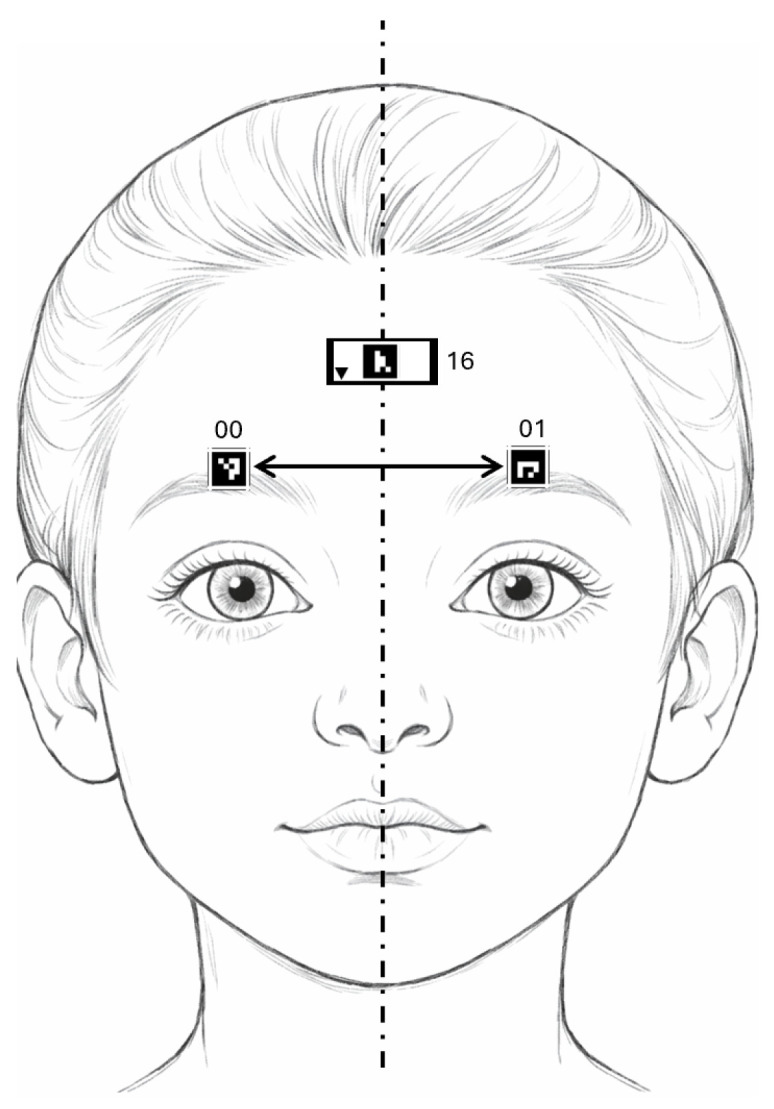
Exercise “Eyebrow raising”—marker placement.

**Figure 3:**
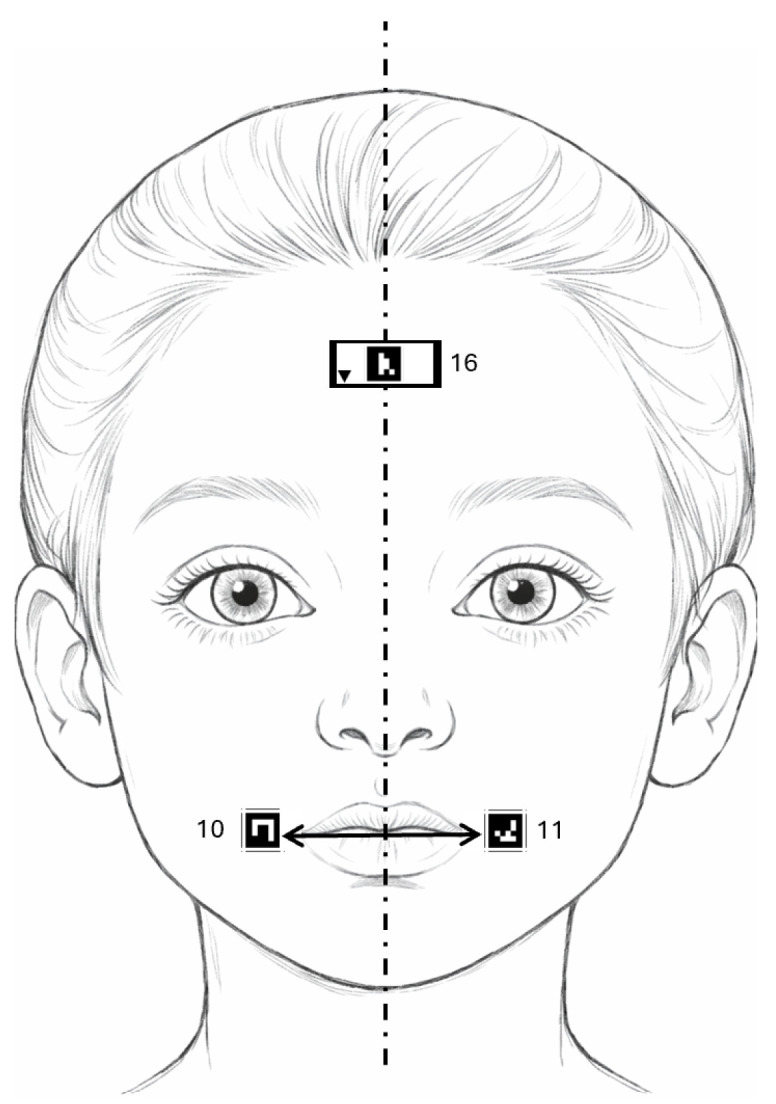
Exercise “Smiling”—marker placement.

For each video frame, distances for the selected markers were computed according to the performed exercise. In the “Eyebrow raising” exercise, we analyzed markers located above the eyebrows (Figure 2), whereas in the “Smiling” exercise, we considered markers positioned near the mouth corners (Figure 3). Distances were measured between each marker and the facial symmetry axis, represented in the figures by a dashed line, with arrows indicating the measured distances. Each marker is additionally labeled with its individual ID number. We supplemented these measurements with additional parameters, including the ratio of distances within marker pairs and the difference in their vertical positions.

#### 2.2.1. Feature Definition

**Figure 4:**
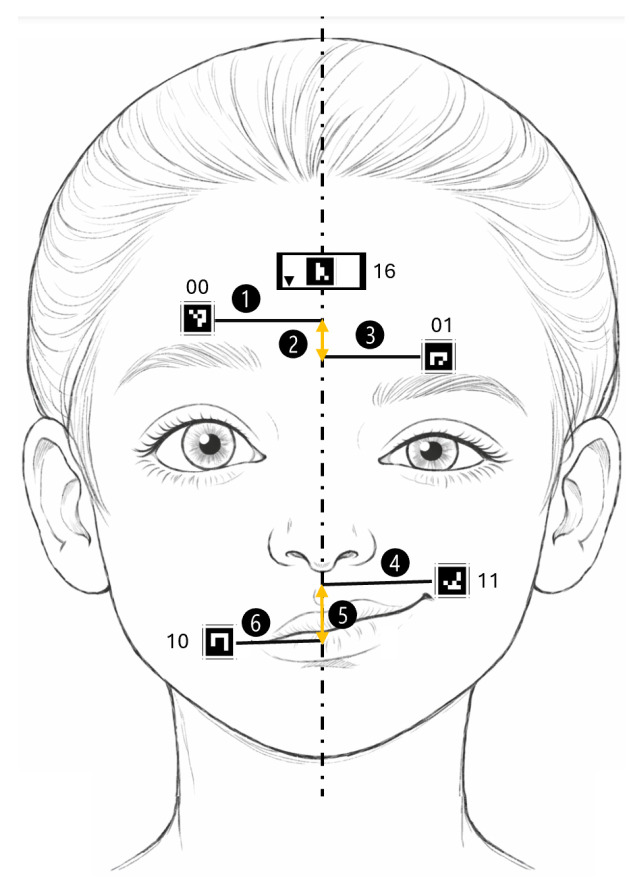
Visualization of parameters: dots labeled as 1, 3, 4 and 6 correspond to marker-symmetry axis distances whereas dots labeled as 2 and 5 to vertical distance (VertDist).

The following features and descriptors were used to parameterize facial movements (Figure 4):

- VertDist—vertical distance between pairs of markers over time (for the “Eyebrow raising” exercise: marker 00 and marker 01; for the “Smiling” exercise: marker 10 and marker 11).
- Ratio—ratio of the distance of a marker on one side of the face from the facial symmetry axis to the corresponding distance of the marker on the opposite side; the value was computed for each video frame.

Let *t* denote the video frame index, and let $p_{i}\left(t\right)=\left(x_{i}\left(t\right),y_{i}\left(t\right)\right)$ denote the coordinates of marker *i* detected in frame *t*. The facial symmetry axis in frame *t* was estimated and represented in the general line form:

(1) a(t)x+b(t)y+c(t)=0,

where (x,y) are image-plane coordinates and a(t), b(t), and c(t) are the coefficients of the axis estimated for frame *t*.

The perpendicular distance between marker *i* and the facial symmetry axis was computed as:

(2) $$d_{i}\left(t\right)=\frac{|a\left(t\right)x_{i}\left(t\right)+b\left(t\right)y_{i}\left(t\right)+c\left(t\right)|}{\sqrt{a^{2}\left(t\right)+b^{2}\left(t\right)}}.$$

For pairs of homologous markers *i* and *j* located on the left and right sides of the face, the vertical inter-marker distance (VertDist) was defined as:

(3) $$VertDist\left(t\right)=\;|y_{i}\left(t\right)-y_{j}\left(t\right)|.$$

The ratio feature (Ratio), describing lateral asymmetry with respect to the facial symmetry axis, was defined as:

(4) $$Ratio\left(t\right)=\frac{d_{L}\left(t\right)}{d_{R}\left(t\right)},$$

where $d_{L}\left(t\right)$ and $d_{R}\left(t\right)$ denote the distances of the left and right markers from the symmetry axis in frame *t*.

Additional descriptors used in the analysis are described in Appendix B.

#### 2.2.2. Physiotherapists Labeling

Three independent experts evaluated each execution of an exercise (“Smiling”, “Eyebrow raising”) performed by a patient. The procedure consisted of unassisted observation of the video recording, which presented the patient performing the exercise. The expert’s task was to assign a label indicating whether the exercise was performed symmetrically or asymmetrically (AS—asymmetric execution, S—symmetric execution). Only functional symmetry, i.e., the movement itself, was assessed, without considering other factors.

Experts evaluated only raw video recordings, without visualization of numerical parameters, to minimize the risk of bias in the assessment. For further analysis, we assigned the label agreed upon by at least two of the three experts (majority rule: 2/3), which served as a pragmatic reference rather than an unequivocal gold standard. Agreement between the three independent experts was assessed using Fleiss’ kappa coefficient. Moderate agreement was obtained for the “Smiling” exercise (κ=0.57) and slightly lower but still moderate agreement for the “Eyebrow raising” exercise (κ=0.47).

#### 2.2.3. Statistical Analysis

After determining the features that describe the movement of the selected markers, we performed statistical analysis. This step aimed to verify whether significant differences occurred between the group of patients performing a given exercise symmetrically and asymmetrically, as measured by the extracted signal features (movement trajectories). For this purpose, we computed statistical descriptors of the signal features. The analysis was conducted separately for each exercise; in each case, patients formed two independent groups. The execution type (S–symmetric/AS–asymmetric) may differ for the same patients depending on the analyzed exercise. Our analysis proceeded in the following stages: we normalized the data and determined signal feature descriptors, including parameters characterizing the distribution and dynamics of changes.

Feature normalization was performed using a median- and median absolute deviation (MAD)-based approach, referred to as a robust z-score. The procedure was carried out independently for each feature and each patient, allowing for a comparison of feature values across patients and minimizing the influence of outliers.

For normalized features, we computed dispersion measures that describe the distribution of values over time, including standard deviation (STD), median absolute deviation (MAD), and interquartile range (IQR). Additionally, to capture temporal dynamics, the first derivative of each feature was calculated, followed by computation of the corresponding dispersion measures (dSTD, dMAD, dIQR). The prefix *d* indicates that the descriptors were calculated for the first derivative of the feature.

The last stage involved verifying hypotheses using statistical tests. Normality of the distributions was assessed using the Shapiro–Wilk test, performed separately for the symmetric (S) and asymmetric (AS) groups for each analyzed descriptor. Homogeneity of variances was evaluated using Levene’s test. Due to unequal group sizes, group comparisons were conducted using Welch’s *t*-test with effect size assessment using Hedges’ g. For group differences (Cohen’s d or Hedges’ g), small, medium, and large effect sizes should correspond to 0.1, 0.4, and 0.8, respectively [20]. Results with a significance level of p<0.05 were considered statistically significant. Statistical analysis was performed using JASP software (version 0.95.4) [21].

In addition to group-level hypothesis testing, we evaluated the univariate discriminative ability of each descriptor to separate symmetric (S) and asymmetric (AS) executions using receiver operating characteristic (ROC) analysis. For each feature, the ROC curve defined by the true positive rate (TPR) and false positive rate (FPR), and the area under the curve (AUC) were computed. The optimal operating point was selected using the Youden index (J=sensitivity+specificity−1).

## 3. Results

This section presents the results of the statistical analysis of movement descriptors, comparing symmetric and asymmetric executions of the analyzed facial exercises.

### 3.1. Statistical Comparison

The analysis included descriptors of the first derivatives of the Ratio and VertDist features. Comparisons were performed between the groups that executed the exercises symmetrically (S) and asymmetrically (AS). Although statistical descriptors were calculated for both the original Ratio and VertDist signals and their first derivatives, statistically significant group differences were found only for the descriptors of the first derivatives.

Additional descriptive statistics and assumption checks are provided in Appendix A.

### 3.2. “Eyebrow Raising” Exercise

**Figure 5:**
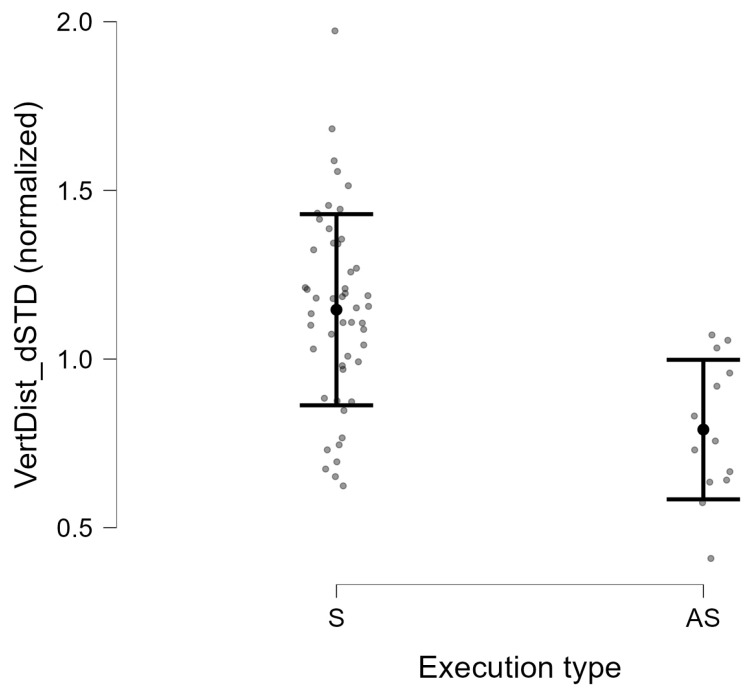
Distribution of the normalized VertDist_dSTD descriptor for symmetric (S) and asymmetric (AS) execution of the eyebrow raising exercise. Grey points represent individual subjects.

For all descriptors based on the first derivative of the VertDist feature, statistically significant differences were observed between the S and AS groups (p<0.001; Table 2). The values of the VertDist_dSTD, VertDist_dMAD, and VertDist_dIQR parameters were higher in the S group. Effect sizes indicated strong discrimination between groups (Hedges’ |g|=1.41−1.42). The group-wise distribution of the VertDist_dSTD descriptor for symmetric and asymmetric execution is shown in Figure 5.

Significant differences between groups were also observed for descriptors derived from the first derivative of the Ratio feature (p<0.05). The parameters Ratio_dSTD, Ratio_dMAD, and Ratio_dIQR reached higher values in the S group, with moderate effect sizes (Hedges’ |g|=0.75−0.87).

**Figure 6:**
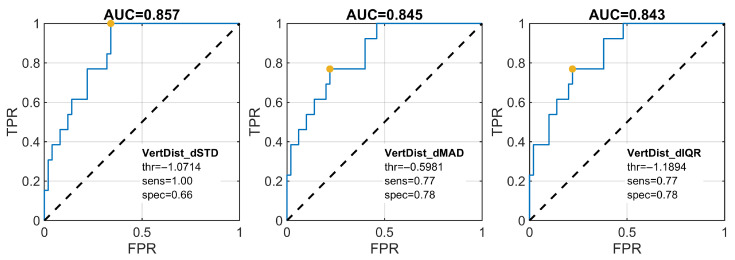
Receiver operating characteristic (ROC) curves for the top three velocity-based features distinguishing AS and S groups during the eyebrow raising exercise. The optimal operating point was determined using the Youden index.

ROC analysis for the eyebrow-raising exercise showed that the three highest AUC values (approximately 0.84–0.86) were obtained for velocity-based descriptors derived from the first derivative of VertDist (Figure 6).

### 3.3. “Smiling” Exercise

**Figure 7:**
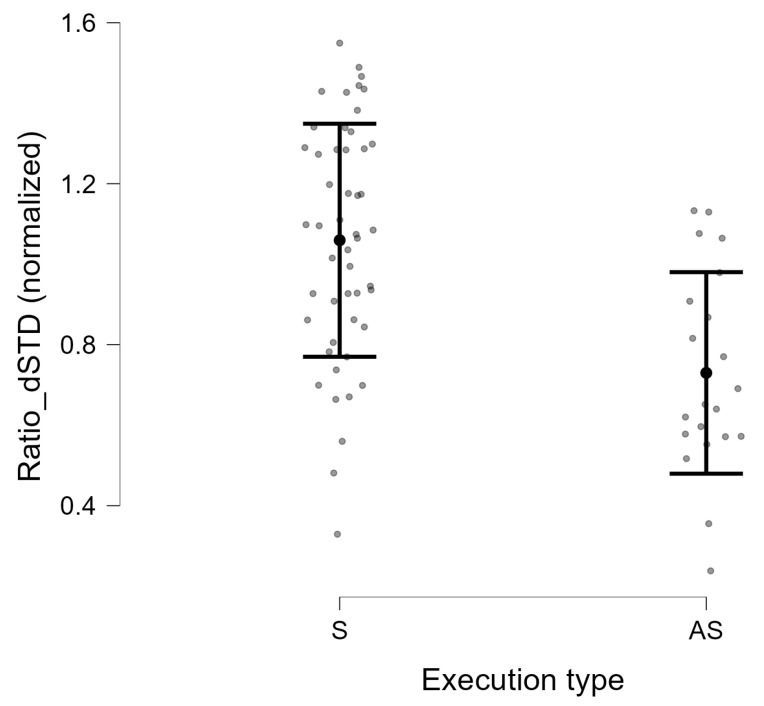
Distribution of the normalized Ratio_dSTD descriptor for symmetric (S) and asymmetric (AS) execution of the smiling exercise. Grey points represent individual subjects.

For all descriptors based on the first derivative of the Ratio feature, we observed statistically significant differences between the S and AS groups (p<0.001; Table 3). The parameters Ratio_dSTD, Ratio_dMAD, and Ratio_dIQR exhibited higher values in the S group, with effect sizes ranging from Hedges’ |g|=1.14−1.21. The distribution of the Ratio_dSTD descriptor for symmetric and asymmetric execution is presented in Figure 7.

Significant differences were also noted for descriptors based on the first derivative of the VertDist feature (p≤0.003). The parameters VertDist_dSTD, VertDist_dMAD, and VertDist_dIQR were higher in the S group, with moderate to large effect sizes (Hedges’ |g|=0.87−0.93).

**Figure 8:**
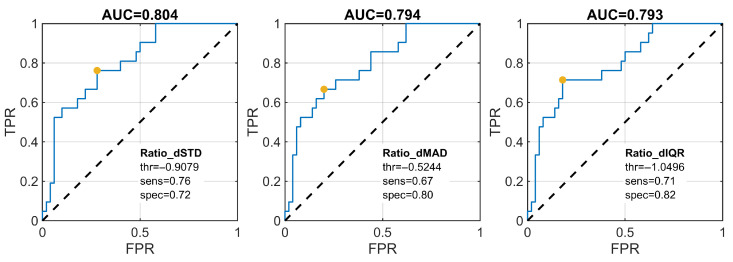
Receiver operating characteristic (ROC) curves for the top three velocity-based features distinguishing AS and S groups during the smiling exercise. The optimal operating point was determined using the Youden index.

For the smiling exercise, ROC curves of the three top-ranked velocity-based descriptors yielded AUC values in a similar range (approximately 0.84–0.86), with operating points determined using the Youden index (Figure 8).

## 4. Discussion

The conducted analysis demonstrated significant differences between symmetric and asymmetric executions in both analysed facial exercises. The most discriminative measures were descriptors based on the first derivatives of the VertDist and Ratio features, indicating that movement dynamics provide informative characteristics for differentiating between execution types. These findings are consistent with previous reports highlighting the relevance of dynamic movement descriptors in facial motion analysis [22,23]. Furthermore, in analysed dataset, asymmetric executions were associated with a narrower range and lower movement variability, reflected in lower values of dynamic descriptors compared to symmetric executions.

In this study, vertical displacement was used as a primary discriminative feature, while horizontal movement was indirectly captured through ratio-based inter-landmark distances along the x-axis. Although full two-dimensional vectorial motion was not explicitly modeled, the proposed feature set enables characterization of the fundamental kinematic aspects of the analyzed expressions while preserving methodological simplicity.

For the eyebrow-raising task, descriptors based on the vertical component of movement (VertDist) showed the greatest discriminative ability. In contrast, for the smiling task, relational descriptors (Ratio) describing dependencies between the two sides of the face more strongly differentiated the groups. For the two analysed tasks, different features demonstrated stronger discriminative ability, highlighting task-specific differences in feature performance. The observed effect sizes, with Hedges’ g values ranging from approximately 0.75 to 1.42, indicate moderate to large differences between groups.

The ROC-based feature ranking indicates that velocity-derived descriptors provide higher univariate separability between symmetric and asymmetric executions than static measures, suggesting that asymmetry in facial expressions is primarily reflected in dynamic movement characteristics. The observed AUC values in the range of approximately 0.84–0.86 indicate a consistent level of separation across exercises.

In contrast to previously reported methods, our study involves patients with actual facial asymmetries, whereas some earlier works employed artificially induced asymmetries in healthy volunteers. In those studies, participants were required to physically stabilize their heads to avoid midline displacement errors. In contrast, our method maintains an accurate estimation of asymmetry despite natural head movements, allowing for dynamic analysis of facial expressions under clinical conditions [24]. Other studies have adopted a longitudinal approach, assessing symmetry changes before surgery and at 6 and 12 months postoperatively. In contrast, our data consist of a single measurement, which limits the ability to track changes over time but supports a rapid estimation of asymmetry within a cross-sectional framework. Despite differences in study designs, both our findings and previous work support the utility of dynamic analysis in capturing functional facial asymmetry [5].

An important component of the proposed method was frame-by-frame estimation of the facial symmetry axis, which served as a local reference frame for the computed parameters and reduced the influence of global head movements. In this context, maintaining consistent localization of the face and eyes across consecutive video frames was crucial, as it enabled stable estimation of the symmetry axis over time while allowing the analysis to be performed with low computational burden. The concept of using a symmetry axis as the basis for quantitative asymmetry assessment is consistent with previous studies in geometric facial analysis [1,25,26]. This solution is particularly useful in pediatric populations, although it does not completely eliminate the influence of head tilt; this issue requires further investigation. The obtained results are consistent with observations related to the perception of facial movement asymmetry, where temporal and amplitude differences between the two sides of the face have been shown to be detectable and functionally relevant [3]. These findings justify the use of objective quantitative methods, given the subjective nature of traditional clinical grading scales [2].

The moderate agreement among expert ratings (κ=0.47–0.57) further confirms the subjectivity of visual assessment and supports the need for objective quantitative approaches. Compared to deep learning models, which may also offer interpretability but typically require additional explainability techniques, the proposed descriptors provide simpler, more direct interpretations [8,27]. The method may serve as a supportive tool for expert assessment and for monitoring rehabilitation progress, both in clinical and home-based settings. Moreover, it aligns with current trends in the development of objective systems for orofacial function assessment using motion tracking techniques [7].

In summary, our results confirm that dynamic descriptors based on the first derivatives of the VertDist and Ratio features effectively differentiate symmetric and asymmetric executions and can be used for quantitative assessment of functional facial movement asymmetry. The results support the hypothesis stated in the introduction that first-derivative dynamic descriptors effectively distinguish between symmetric and asymmetric executions.

This study has several limitations. The analysis was restricted to two facial exercises and involved a limited sample size, with unequal numbers of symmetric and asymmetric executions. The analysis was based on 2D video data without full 3D head-pose compensation, and the facial symmetry axis was determined solely from eye positions. Moreover, ROC and AUC analyses were used to quantify univariate feature separability rather than to construct a predictive classifier. As such, the reported operating points should be interpreted descriptively, and future work should address multivariate modeling and validation.

Although the proposed approach shows promise, the present study represents an initial methodological exploration rather than a validated clinical tool. At this stage, no data are available regarding diagnostic sensitivity or specificity, normative population values, or correlations with established clinical outcome measures. Consequently, any potential clinical applications such as diagnostic use, therapy planning, or inter-centre comparisons should be considered preliminary. Further studies involving larger and clinically diverse cohorts, standardized reference measures, and longitudinal validation are required before clinical implementation can be justified.

An additional limitation of the present study is its reliance on a single cross-sectional measurement. While this allows for the identification of asymmetry at a given time point, it is not methodologically comparable to studies designed to track temporal changes or intervention-related effects. The absence of a longitudinal component substantially limits functional interpretation and precludes conclusions regarding progression, adaptation, or treatment response. Future longitudinal studies are necessary to assess the stability and clinical relevance of the observed asymmetry over time. These limitations indicate that the results should be generalized with caution.

## 5. Conclusions

In this paper, we proposed a low-cost marker-based video method that enables an objective description of functional facial movement asymmetry. Dynamic descriptors derived from the first derivatives of the VertDist and Ratio features significantly differentiated between symmetric and asymmetric executions, confirming the study’s hypothesis. The method shows promise for objective assessment of facial movement; however, its use in clinical or home-based rehabilitation remains to be validated. Future work should include additional facial tasks, larger cohorts, and long-term follow-up studies.

## Figures and Tables

**Table 1:** Number of patients included in the analysis with basic demographic characteristics (AS—asymmetric execution, S—symmetric execution).

Exercise	AS	S	Age [Years] (Mean ± SD)
Eyebrow raising	13	50	14 ± 4
Smiling	21	50	14 ± 4

**Table 2:** Comparison of dynamic descriptors of eyebrow movement between S and AS groups using Welch’s *t*-test.

Descriptor	*t*	df	*p*	Hedges’ *g*
VertDist_dSTD	−5.077	25.10	<0.001	−1.414
VertDist_dMAD	−4.817	21.48	<0.001	−1.410
VertDist_dIQR	−4.851	21.69	<0.001	−1.415
Ratio_dSTD	−2.324	16.80	0.033	−0.747
Ratio_dMAD	−2.746	17.29	0.014	−0.872
Ratio_dIQR	−2.728	17.17	0.014	−0.869

Note. *t*—test statistic of Welch’s *t*-test; df—degrees of freedom estimated using the Welch–Satterthwaite equation; Hedges’ *g*—bias-corrected effect size (negative values indicate higher scores in the S group).

**Table 3:** Comparison of dynamic descriptors of smiling movement between S and AS groups using Welch’s *t*-test.

Descriptor	*t*	df	*p*	Hedges’ *g*
Ratio_dSTD	−4.828	43.21	<0.001	−1.205
Ratio_dMAD	−4.574	42.61	<0.001	−1.145
Ratio_dIQR	−4.562	42.79	<0.001	−1.141
VertDist_dSTD	−3.526	46.34	<0.001	−0.866
VertDist_dMAD	−3.783	46.86	<0.001	−0.928
VertDist_dIQR	−3.756	46.89	<0.001	−0.921

Note. *t*—test statistic of Welch’s *t*-test; df—degrees of freedom estimated using the Welch–Satterthwaite equation; Hedges’ *g*—bias-corrected effect size (negative values indicate higher scores in the S group).

## Data Availability

The data presented in this study are available on request from the corresponding author. The data are not publicly available due to privacy and ethical restrictions.

## Appendix A. Additional Statistical Results

This appendix presents descriptive statistics and assumption checks for dynamic facial movement descriptors across the analyzed facial exercises.

### Appendix A.1. “Eyebrow Raising” Exercise

Descriptive statistics for the “Eyebrow raising” exercise are reported in Table A1. The results of the Shapiro–Wilk normality tests performed separately for the symmetric (S) and asymmetric (AS) groups are presented in Table A2, while the results of the homogeneity of variance assessment using Levene’s test are summarized in Table A3.

**Table A1:** Descriptive statistics of dynamic facial movement descriptors for the eyebrow raising exercise.

Descriptors	Group	*N*	Mean	SD
VertDist_dSTD	AS	13	0.791	0.207
	S	50	1.146	0.283
VertDist_dMAD	AS	13	0.497	0.149
	S	50	0.729	0.176
VertDist_dIQR	AS	13	0.993	0.298
	S	50	1.461	0.354
Ratio_dSTD	AS	13	0.881	0.372
	S	50	1.142	0.317
Ratio_dMAD	AS	13	0.549	0.205
	S	50	0.721	0.183
Ratio_dIQR	AS	13	1.099	0.413
	S	50	1.441	0.364

**Table A2:** Normality assessment of dynamic descriptors for the eyebrow raising exercise using the Shapiro–Wilk test, performed separately for the symmetric (S) and asymmetric (AS) groups.

Descriptor	Group	*N*	*W*	*p*
VertDist_dSTD	AS	13	0.950	0.601
	S	50	0.980	0.532
VertDist_dMAD	AS	13	0.957	0.710
	S	50	0.983	0.704
VertDist_dIQR	AS	13	0.962	0.783
	S	50	0.982	0.630
Ratio_dSTD	AS	13	0.899	0.129
	S	50	0.965	0.146
Ratio_dMAD	AS	13	0.911	0.191
	S	50	0.982	0.621
Ratio_dIQR	AS	13	0.912	0.193
	S	50	0.983	0.696

**Table A3:** Homogeneity of variance assessment for dynamic descriptors in the eyebrow raising exercise using Levene’s test.

Descriptor	*F*	${df}_{1}$	${df}_{2}$	*p*
VertDist_dSTD	0.678	1	61	0.413
VertDist_dMAD	0.346	1	61	0.559
VertDist_dIQR	0.402	1	61	0.528
Ratio_dSTD	0.222	1	61	0.639
Ratio_dMAD	0.143	1	61	0.707
Ratio_dIQR	0.178	1	61	0.674

### Appendix A.2. “Smiling” Exercise

Descriptive statistics for the “Smiling” exercise are presented in Table A4. Normality assessment results obtained using the Shapiro–Wilk test for both groups are reported in Table A5, and the corresponding homogeneity of variance results based on Levene’s test are shown in Table A6.

**Table A4:** Descriptive statistics of dynamic facial movement descriptors for the smiling exercise.

Descriptors	Group	*N*	Mean	SD
Ratio_dSTD	AS	21	0.730	0.250
	S	50	1.060	0.290
Ratio_dMAD	AS	21	0.471	0.167
	S	50	0.677	0.190
Ratio_dIQR	AS	21	0.943	0.335
	S	50	1.358	0.383
VertDist_dSTD	AS	21	0.613	0.209
	S	50	0.820	0.260
VertDist_dMAD	AS	21	0.380	0.132
	S	50	0.520	0.165
VertDist_dIQR	AS	21	0.763	0.262
	S	50	1.040	0.329

**Table A5:** Normality assessment of dynamic descriptors for the smiling exercise using the Shapiro–Wilk test, performed separately for the symmetric (S) and asymmetric (AS) groups.

Descriptor	Group	*N*	*W*	*p*
Ratio_dSTD	AS	21	0.949	0.327
	S	50	0.973	0.304
Ratio_dMAD	AS	21	0.957	0.462
	S	50	0.971	0.251
Ratio_dIQR	AS	21	0.956	0.447
	S	50	0.971	0.260
VertDist_dSTD	AS	21	0.948	0.315
	S	50	0.987	0.853
VertDist_dMAD	AS	21	0.954	0.410
	S	50	0.984	0.717
VertDist_dIQR	AS	21	0.955	0.426
	S	50	0.985	0.779

**Table A6:** Homogeneity of variance assessment for dynamic descriptors in the smiling exercise using Levene’s test.

Descriptor	*F*	${df}_{1}$	${df}_{2}$	*p*
Ratio_dSTD	0.668	1	69	0.417
Ratio_dMAD	0.693	1	69	0.408
Ratio_dIQR	0.747	1	69	0.390
VertDist_dSTD	0.392	1	69	0.533
VertDist_dMAD	0.506	1	69	0.479
VertDist_dIQR	0.503	1	69	0.480

## Appendix B. Additional Feature Descriptors

In addition to the parameters described in the main part of the paper, additional descriptors of the extracted signal features were also computed. These included, among others, skewness, kurtosis, the quantile-based asymmetry coefficient AsymQ calculated from selected quantiles, and entropy estimated from a normalized probability histogram. Selected quantiles (e.g., Q10–Q90) were also analyzed. All measures were computed individually for each patient using a uniform signal processing and normalization procedure.

The statistical analysis of these additional descriptors did not reveal statistically significant differences between groups; therefore, they were not included in the Section 3.
